# The effects of acute stress exposure on striatal activity during Pavlovian conditioning with monetary gains and losses

**DOI:** 10.3389/fnbeh.2014.00179

**Published:** 2014-05-22

**Authors:** Andrea H. Lewis, Anthony J. Porcelli, Mauricio R. Delgado

**Affiliations:** ^1^Department of Psychology, Rutgers UniversityNewark, NJ, USA; ^2^Department of Psychology, Marquette UniversityMilwaukee, WI, USA

**Keywords:** acute stress, cold pressor, conditioning, striatum, fMRI, cortisol

## Abstract

Pavlovian conditioning involves the association of an inherently neutral stimulus with an appetitive or aversive outcome, such that the neutral stimulus itself acquires reinforcing properties. Across species, this type of learning has been shown to involve subcortical brain regions such as the striatum and the amygdala. It is less clear, however, how the neural circuitry involved in the acquisition of Pavlovian contingencies in humans, particularly in the striatum, is affected by acute stress. In the current study, we investigate the effect of acute stress exposure on Pavlovian conditioning using monetary reinforcers. Participants underwent a partial reinforcement conditioning procedure in which neutral stimuli were paired with high and low magnitude monetary gains and losses. A between-subjects design was used, such that half of the participants were exposed to cold stress while the remaining participants were exposed to a no stress control procedure. Cortisol measurements and subjective ratings were used as measures of stress. We observed an interaction between stress, valence, and magnitude in the ventral striatum, with the peak in the putamen. More specifically, the stress group exhibited an increased sensitivity to magnitude in the gain domain. This effect was driven by those participants who experienced a larger increase in circulating cortisol levels in response to the stress manipulation. Taken together, these results suggest that acute stress can lead to individual differences in circulating cortisol levels which influence the striatum during Pavlovian conditioning with monetary reinforcers.

## Introduction

Pavlovian conditioning, a fundamental learning mechanism, involves the acquisition of an association between a neutral conditioned stimulus (CS) and an unconditioned stimulus (US) such that the CS acquires the properties of the US. Numerous laboratory studies have examined the behavioral and neural bases of this process in the absence of external environmental factors, highlighting the involvement of structures such as the striatum, amygdala and medial prefrontal cortex during acquisition (for review see Everitt and Robbins, 2005; Phelps and LeDoux, 2005; Peters et al., 2009; Schiller and Delgado, 2010). While this phenomenon has been well-characterized in the literature, less is known about how inherently negative states, such as those induced by stress, can impact the neural circuits underlying the acquisition of Pavlovian contingencies. Acute stress has been shown to potentiate negative affect and activity in the striatum, further correlating with increased craving in addicted populations (Sinha et al., 2005). Thus, it is important to consider the influence of stress on the formation of simple conditioned responses that can lead to maladaptive behaviors. The goal of the current study was to examine the effect of acute stress on the neural correlates of Pavlovian conditioning using monetary gains and losses.

Stress can affect many basic learning processes on both behavioral and neural levels (see Shors, 2004 for review), but efforts to understand the specific influence of stress on conditioning have produced variable results. The acquisition of a conditioned response during aversive learning in rodents, for example, has been shown to be enhanced in males under stress (Wilson et al., 1975; Shors et al., 1992) and depressed in females under stress (e.g., Wood and Shors, 1998; Wood et al., 2001). This is highly context dependent, however, as factors such as stressor type (e.g., swim stress, noise or restraint; Shors, 2001) and the temporal proximity of the learning process to the experienced stress (see Joëls et al., 2006 for review) can affect the manner in which stress alters learning.

Similar variability in the effects of stress on learning has been observed in humans. For instance, acute stress has been suggested to improve performance in an eyeblink conditioning task in some studies (e.g., Duncko et al., 2007) while also impairing eyeblink conditioning in other reports (e.g., Wolf et al., 2009). One explanation for this discrepancy may be the use of alternative stressors associated with different patterns of cortisol release (e.g., Cold Pressor Test vs. Trier Social Stressor Test). In order to accurately assess the effects of stress on Pavlovian conditioning, it may prove critical to not simply examine differences between participants who were exposed to a stress procedure and those who were not, but also to examine, within participants exposed to stress, individual differences in levels of circulating cortisol. Increased levels of cortisol during fear conditioning in males but not females, for example, correlate with elevated fear acquisition (Zorawski et al., 2005, 2006). It has been suggested that both cortisol levels (Stark et al., 2006; Merz et al., 2013a) and sex (Stark et al., 2006; Merz et al., 2013b) may play a role in determining the specific effects of stress on the processing of CS associated with aversive primary reinforcers.

With respect to the human brain, fMRI studies have observed an influence of stress on activity in associative learning-related brain regions such as the striatum, anterior cingulate cortex, hippocampus and amygdala (Merz et al., 2013b). The striatum, in particular, appears to be vulnerable to the effects of stress (e.g., Sinha et al., 2005; Porcelli et al., 2012), which could subsequently impact learning. Increasing stress by threat of shock, for example, has been found to increase aversive prediction errors during probabilistic learning (Robinson et al., 2013), consistent with observations of aversive prediction errors in the striatum during fear conditioning studies (Seymour et al., 2004; Delgado et al., 2008).

In the current study, participants underwent either an acute stress or no stress procedure, and then performed a simple Pavlovian conditioning task, where neutral shapes were associated with monetary gains and losses of varying magnitudes. Money, a secondary reinforcer, was chosen for use in the study given that it can be somewhat equated in the positive and negative domains (i.e., it can be gained or lost, unlike many primary reinforcers). Neuroimaging studies in humans have highlighted a role for the striatum in Pavlovian conditioning with appetitive and aversive primary reinforcers (e.g., juice: O'Doherty et al., 2004; shock: see Phelps and LeDoux, 2005, for review), and also with secondary reinforcers (e.g., money: Kirsch et al., 2003; Valentin and O'Doherty, 2009; Delgado et al., 2011). Thus, we hypothesized that the striatum would be engaged in our simple Pavlovian conditioning paradigm with secondary reinforcers. Additionally, we used a modified version of the cold pressor test as our stressor (Porcelli, 2014), previously shown to induce stress responses in a between-subjects design (Porcelli et al., 2012). We hypothesized that during Pavlovian conditioning, acute stress would modulate neural activity in regions typically involved in associative learning, such as the striatum.

## Materials and methods

### Participants

Thirty-four individuals were recruited via flyers posted on the Rutgers campus. All participants were right handed and were prescreened for MRI contraindications. Participants did not have a psychiatric disorder at the time of the experiment, were not taking psychotropic medications at the time of the experiment, and were not under the care of a psychologist or psychiatrist at the time of the experiment. Information on menstrual cycle phase was collected for 13 out of 16 female participants. Three female participants were tested during the luteal phase and 10 were tested during the follicular phase. Two participants were excluded from final data analysis, one due to an MRI equipment failure and the other resulting from a request to withdraw from participation. Thus, final data analysis was performed on 32 participants (16 females, 16 males; mean age = 23.41 years, *SD* = 4.07). All participants gave informed consent according to the guidelines of the Institutional Review Boards of Rutgers University and the University of Medicine and Dentistry of New Jersey.

### Procedure

**Figure 1:**
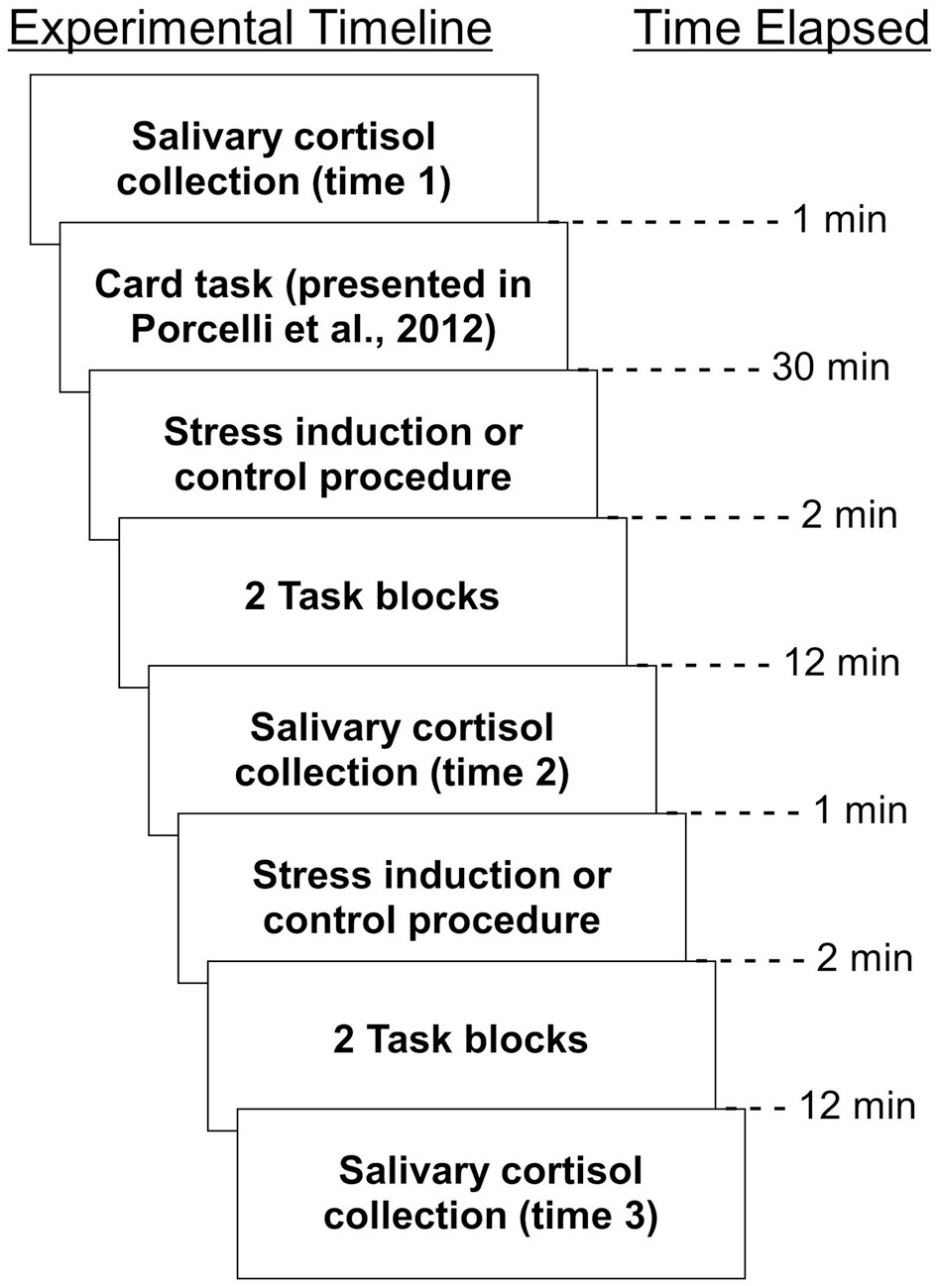
**Timeline of experimental activities**. All procedures, as well as time elapsed between them, are depicted.

Experimental sessions were conducted between the hours of 1:00 pm and 5:30 pm to account for circadian fluctuations in cortisol (Kirschbaum and Hellhammer, 1994). In order to obtain informed consent, all participants were told about the nature of the stress procedure. That is, participants were initially informed that they would be exposed to either a stress or no stress control procedure in a between-subjects fashion, but were not assigned to the stress group or no stress group until the start of the experiment. Participants were notified that they could withdraw from the study at any time. All participants completed the experimental activities described in Porcelli et al. (2012), and immediately afterward, remained in the scanner to participate in a simple learning task during which monetary rewards could be earned. Participants underwent the following procedure: (1) salivary cortisol collection, (2) stress induction or control procedure, (3) two conditioning task runs, (4) salivary cortisol collection, (5) stress induction or control procedure, (6) two conditioning task runs, (7) salivary cortisol collection. Each procedure is described in detail below. A timeline of experimental procedures are outlined in Figure 1.

### Stress induction

We utilized a variant on the traditional cold pressor test (Lovallo, 1975), which involves immersion of one's hand into a container of ice-cold water, prior to the first and third conditioning task runs. We chose to use the cold pressor task as it has been used with success as an acute stressor in previous studies (e.g., Ferracuti et al., 1994; Cahill et al., 2003; Ishizuka et al., 2007; Porcelli and Delgado, 2009). It is important to note that although water is not inherently incompatible with the MRI environment, if spilled it can be a threat to sensitive MRI equipment (such as the head coil). Additionally, even in the absence of damage due to a spill water can interfere with MRI signal due to its high proton density (Huettel et al., 2008). In the current experiment, we utilized a modified cold pressor test that is compatible with the MRI environment (Porcelli et al., 2012; Porcelli, 2014). To administer cold pressor stress safely once participants were placed within the MRI, rather than prior to entry, an arm wrap was created from a combination of MRI-compatible dry gelpacs and maintained at a temperature of approximately 4°C. This “cold pressor arm wrap” was placed around the right hand and arm of participants assigned to the acute stress group for 2 min prior to prior to the first and third conditioning task runs. The cold pressor arm wrap, which has been shown to successfully increase sympathetic nervous system activation (Porcelli, 2014), was applied at approximately 15 min intervals throughout the course of the experiment. Stress was administered twice during the experimental activities presented in Porcelli et al. (2012), and twice during the current study. The frequent administration of stress was intended to allow for the maintenance of increased sympathetic nervous system activation. Participants were told both during consent procedures as well as immediately prior to the MRI experiment that they could have the cold pressor arm wrap removed at any time by calling the experimenter. For participants assigned to the no stress group, a similar wrap created from room-temperature towels was applied to control for tactile stimulation of the cold pressor arm wrap.

Although cold stress has been applied for 3 min in some studies, it has also been applied for 2 min in others (e.g., Olga et al., 1995; Schobel et al., 1995; Busjahn et al., 1996; O'Sullivan and Bell, 2001; Kuniyoshi et al., 2003; Porcelli et al., 2008, 2012; Porcelli and Delgado, 2009). Other studies have even used a 1 min exposure (e.g., Maekawa et al., 1999; Duncko et al., 2007, 2009). It is also notable that immersion of 3 min is a maximum time, and participants vary in how long they allow their hand to remain immersed (e.g., Mitchell et al., 2004).

At the end of the experiment, participants were asked to rate how stressful they found the stress or control procedure and how the cold pressor or control arm wrap made them feel. Specifically, the questions “How much stress did you feel from the cold procedure (or room temperature procedure)?” and “How did exposure to the cold arm wrap (or room temperature arm wrap) make you feel?” were asked, and participants answered each question by selecting a number from 1 to 7 on a Likert scale.

### Salivary cortisol measurements

Participants were instructed to avoid eating, drinking (anything other than water), or smoking for 2 h prior to the beginning of the experiment to ensure that saliva samples were untainted. To acquire salivary cortisol data, participants were asked to moisten a Salimetrics Oral Swab (SOS) in their mouths for 1 min by placing the SOS underneath their tongue. Upon completion of this procedure, the participant withdrew the SOS and the experimenter immediately placed it in an individual centrifuge tube. Three samples were acquired for each participant. The first (baseline) sample was acquired at the start of all experimental procedures [that is, immediately preceding the experimental activities described in Porcelli et al. (2012)]. Importantly, the first sample was not acquired until after the anatomical MRI scans, which lasted approximately 15 min, had been completed. This allowed participants sufficient time to acclimate to the scanner environment. The second sample was acquired prior to the third conditioning task run, and the third sample was acquired at the end of the experiment, immediately following the fourth conditioning task run. Samples were frozen in cold storage at −10°C, packed with dry ice and sent to Salimetrics Laboratory (State College, PA) for duplicate biochemical assay analysis.

### Conditioning task

**Figure 2:**
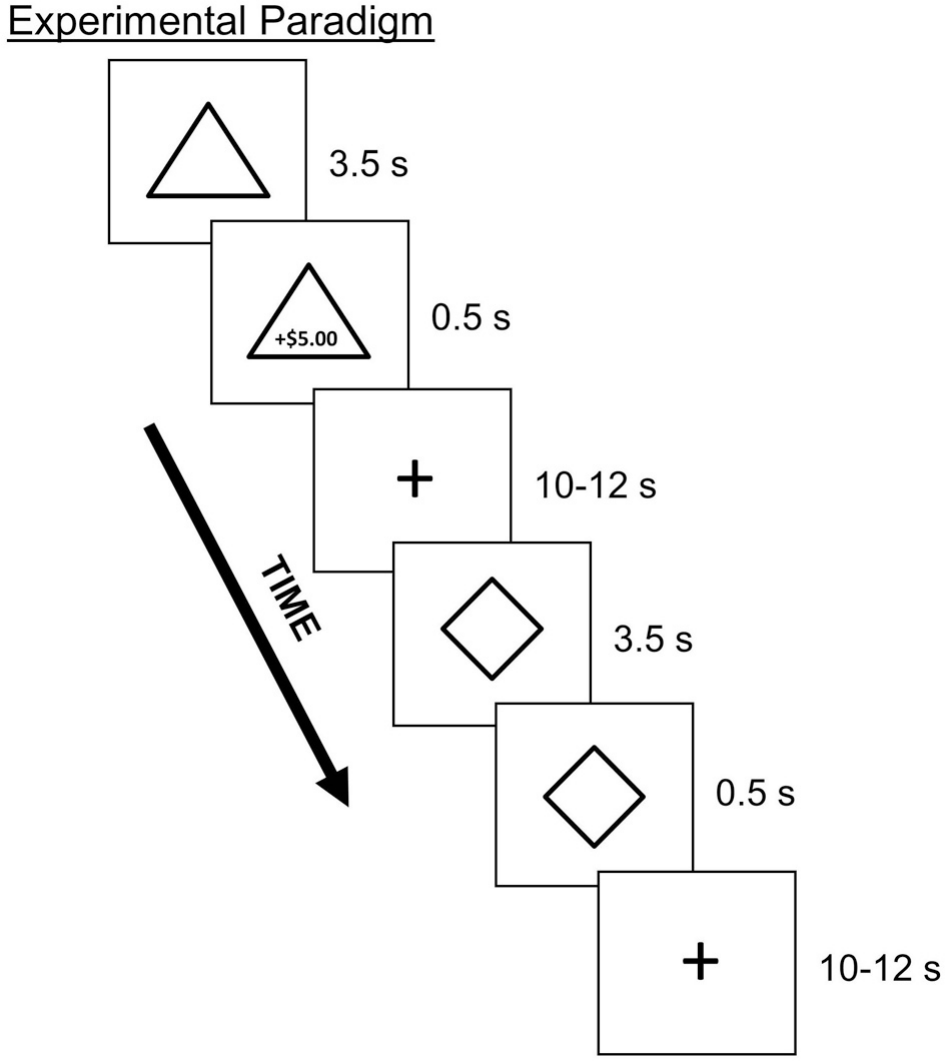
**Depiction of the Pavlovian conditioning task with partial reinforcement**. On each trial, participants viewed a geometric shape (CS; e.g., triangle, diamond) that was paired with a monetary outcome (US; e.g., $5.00 gain) 50% of the time. Stimuli consisted of four different shapes, each paired with a specific monetary outcome that varied with respect to valence and magnitude.

Participants underwent four runs of a Pavlovian conditioning task during which they learned associations between neutral CS and monetary US (Figure 2). On each trial, a geometric shape (CS) appeared for 4 s. At 3.5 s following CS onset, text indicating the amount of monetary gain or loss (US) appeared, with duration of 0.5 s. Both the CS and US co-terminated at 4 s. Trials were separated by a jittered inter-trial interval of either 10 or 12 s. Each CS was paired with a specific US which varied with respect to valence (i.e., monetary gain or loss) and magnitude (i.e., high or low value). The four potential monetary outcomes were as follows: gains, +$5.00 or +$0.50; losses, −$2.50 or −$0.25. Monetary losses were of lower magnitude than the monetary gain of corresponding magnitude in order to account for loss aversion (Tversky and Kahneman, 1991) as has been used in prior studies in our lab (e.g., Delgado et al., 2000). Within a run, participants viewed six of each trial type (counterbalanced), for a total of 24 trials per run. A partial reinforcement procedure was used such that each CS was reinforced either with a US or no US 50 percent of the time. Therefore, within each run, a total of 12 trials were reinforced with either monetary gain or loss. Importantly, participants were told that at the end of the experiment, they would receive bonus money equivalent to the amount gained in the conditioning task.

Following each run of the conditioning task, participants rated the four CS on a 5-point Likert scale, ranging from “strongly dislike” to “strongly like.” Given that the conditioning task was Pavlovian in nature, these affective ratings functioned as a behavioral measure of learning.

### fMRI acquisition and analysis

Images were collected using a 3 T Siemens Allegra scanner equipped with a fast gradient system for echoplanar imaging. Structural images were collected using a T1-weighted MPRAGE sequence (256 × 256 matrix; FOV = 256 mm; 176.1 mm sagittal slices). Functional images were acquired using a single-shot gradient echo EPI sequence (*TR* = 2000 ms, *TE* = 25 ms, FOV = 192, flip angle = 80°, bandwidth = 2604 Hz/Px, echo spacing = 44) and comprised 32 contiguous oblique-axial slices (3 × 3 × 3 mm voxels) parallel to the anterior commissure-posterior commissure line. Functional images were collected during all four runs of the conditioning task. BrainVoyager QX software (version 2.2, Brain Innovation, Maastricht, The Netherlands) was used to preprocess and analyze the imaging data. Preprocessing consisted of 3D motion correction (six parameters), slice scan time correction (cubic spline interpolation), spatial smoothing with a 3D Gaussian filter (4 mm FWHM), voxelwise linear detrending, and high-pass filtering of frequencies (three cycles per time course). Structural and functional data for each participant were then transformed to standard Talairach stereotaxic space (Talairach and Tournoux, 1988).

In modeling the conditioning task, a random effects general linear model (GLM) was conducted using each of the four CS as regressors of interest. Thus, both the valence and magnitude of the outcome associated with each CS were accounted for: high magnitude gain, low magnitude gain, high magnitude loss, low magnitude loss. In order to ensure that our model accounted for CS presentation alone, and not US presentation, only the first 2 s of each CS were modeled. We also included six regressors of no interest (six motion parameters). Regressors were convolved with a 2-gamma hemodynamic response function and z-transformed at the single participant level. Correction for multiple comparisons was verified using the Cluster Level Statistical Threshold Estimator plugin in BrainVoyager (Forman et al., 1995; Goebel et al., 2006). This correction method runs a series of Monte Carlo simulations across the whole brain to determine the probability that observed significant clusters of activation are not false positives in a given statistical parametric map. After correction, the map applies the minimum cluster size threshold that produces the cluster level false positive alpha rate (5%). Resulting statistical maps were set to a threshold of *p* < 0.005 and corrected to a whole brain clusters correction threshold of *p* < 0.05 with a threshold of five contiguous voxels (135 mm^3^ as determined by the plugin). All *post-hoc* analyses consisting of more than two *t*-tests within a family of comparisons were corrected for multiple comparisons with the sequential Bonferroni correction (Holm, 1979; Rice, 1989).

### Behavioral analysis

Subjective stress ratings made by the stress and no stress groups were compared through the use of independent *t*-tests. Behavioral ratings from the conditioning task were examined with the use of mixed ANOVAs. All *post-hoc* analyses consisting of more than two *t*-tests within a family of comparisons were corrected for multiple comparisons with the sequential Bonferroni correction (Holm, 1979; Rice, 1989).

## Results

### Subjective stress ratings

Post-experimental subjective ratings of perceived stress experience were examined between acute stress and no stress experimental groups via planned independent *t*-tests. Compared to the no stress group, the acute stress group rated the arm wrap as feeling significantly worse [*t*_(30)_ = 4.42, *p* < 0.001] and the experience as being more stressful [*t*_(30)_ = 3.46, *p* < 0.01]. Males and females did not significantly differ in how the arm wrap made them feel [*t*_(30)_ = 0.11, *p* > 0.05] or in how stressful they found the experience [*t*_(30)_ = 0.33, *p* > 0.05].

### Salivary cortisol data

Salivary cortisol data were excluded for three participants, in one case due to a corruption of the samples and in two cases due to an inability to acquire samples during MRI scanning. Thus, cortisol analyses were conducted on 29 of the 32 participants (13 no stress, 16 acute stress). Female participants were screened for use of oral contraceptives that might influence cortisol levels (though information was not used as an exclusionary criterion *per se*). Five of the sixteen female participants did report use of oral contraceptives, however no significant differences in cortisol levels were observed between female participants using oral contraceptives and those not using oral contraceptives as measured by repeated-measures ANOVA [*F*_(1, 12)_ = 0.365; *p* = 0.557]. Mean and standard deviation of cortisol levels for males and females in the stress and no stress groups are reported in Table 1.

**Table 1:** **Cortisol levels (in μg/dL, mean and standard deviation) for males and females in the stress and no stress groups**.

**Group**	**Baseline**		**Time 1**		**Time 2**	
	**Mean**	***SD***	**Mean**	***SD***	**Mean**	***SD***
Males—stress	0.150	0.042	0.146	0.094	0.139	0.074
Males—no stress	0.156	0.068	0.117	0.038	0.124	0.044
Females—stress	0.125	0.037	0.133	0.053	0.115	0.066
Females—no stress	0.127	0.069	0.137	0.095	0.138	0.097

Area under the curve with respect to increase (AUC_I_) was calculated using the trapezoidal method for both experimental groups (Pruessner et al., 2003). A one-tailed independent *t*-test between AUC_I_ for the experimental groups (stress vs. no stress) was not significant, but was approaching a trend toward increased AUC_I_ for stress group participants, [*t*_(27)_ = 1.303, *p* = 0.102]. Males and females did not significantly differ in AUC_I_ measurements [*t*_(27)_ = 1.016, *p* = 0.319]. Additionally, one-tailed *t*-tests between experimental groups (stress vs. no stress) were conducted separately for males and females. For both males and females, there was no significant difference in AUC_I_ between stress and no stress participants [males, *t*_(13)_ = 1.107, *p* = 0.144; females, *t*_(12)_ = 0.578, *p* = 0.287].

As discussed in the methods, this study was completed immediately after an unrelated task that also involved acquisition of three saliva samples, the third of which was acquired immediately prior to the start of the current study. When a similar AUC_I_ analysis was performed on those three samples alone, a significant increase in cortisol levels was observed for participants exposed to acute stress, *t*_(27)_ = 1.78, *p* < 0.05 (one-tailed). Thus, the stress induction was successful over the course of the scanning session. A similar approach, in which a stress induction was deemed successful at the end of the first task in a two-study experimental session, has been used in other stress research (e.g., Mather et al., 2010).

**Figure 3:**
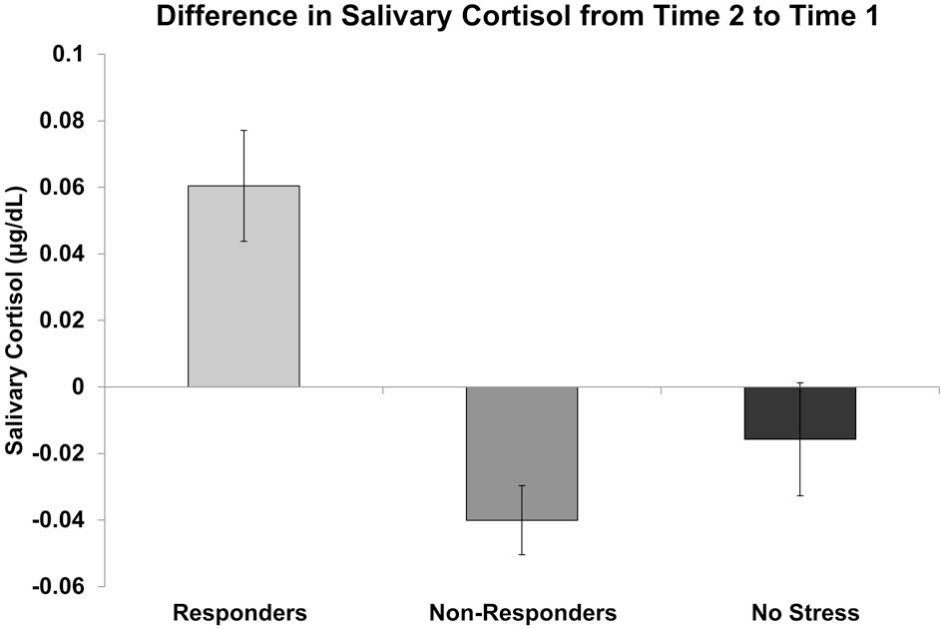
**Difference in salivary cortisol levels (μg/dL) from time 2 (prior to the third conditioning task run) to time 1 (baseline) for cortisol responders, non-responders, and no stress participants**.

The lack of a significant difference in AUC_I_ between the stress and no stress group samples acquired after the current study began may be attributed in part to the length of time participants had been in the MRI. Additionally, some participants in the stress group did not show elevated cortisol responses despite showing high subjective ratings of stress. Given both of these possibilities, as well as the fact that we were interested in examining the effects of increased cortisol on Pavlovian conditioning following the stress manipulation, rather than treating the stress group as homogenous, we divided it into two subgroups: cortisol responders (“responders”) and cortisol non-responders (“non-responders”). To do this, cortisol levels at time 2 (post initial stress exposure but prior to the third conditioning task run) were compared to cortisol levels at time 1 (baseline) for each participant. Using a criterion suggested by Miller et al. (2013), those participants who showed a cortisol increase of at least 15.5% from baseline were placed in the responder group (*n* = 7, four females, mean age = 25.43, *SD* age = 3.60, mean increase = 0.060 μg/dL, *SD* increase = 0.044) while those who did not show an increase of at least 15.5% from baseline were placed in the non-responder group (*n* = 9, four females, mean age = 21.56, *SD* age = 2.65, mean increase = −0.040 μg/dL, *SD* increase = 0.031). Differences in salivary cortisol from time 2 to time 1 for responders, non-responders and no stress participants are displayed in Figure 3.

### Behavioral ratings

At the end of each of the four conditioning runs, participants were asked to rate each of the four CS on a five-point Likert scale ranging from “strongly dislike” to “strongly like.” All participants correctly learned CS-US associations by the end of the first conditioning run, as measured by the following criterion: [(high gain CS rating + low gain CS rating) − (high loss CS rating + low loss CS rating)] > 0. All participants met this criterion at the end of all four conditioning runs. Across all four runs, average behavioral ratings were as follows: high gain, mean = 3.98, *SD* = 0.061; low gain, mean = 3.26, *SD* = 0.40; high loss, mean = 1.03, *SD* = 0.11; low loss, mean = 1.69, *SD* = 0.42. Behavioral ratings for males and females did not differ for any of the four CS (all *p*'s > 0.05).

Collapsed across all runs, a 2 (CS valence) × 2 (CS magnitude) × 2 (stress group) mixed ANOVA was run, and revealed a main effect of valence [*F*_(1, 30)_ = 1075.64; *p* < 0.001], wherein CS associated with monetary gains were rated as significantly higher than CS associated with monetary loss. A valence × magnitude interaction was also observed [*F*_(1, 30)_ = 95.69; *p* < 0.001]. No main effect of stress group was observed (all *p*'s > 0.05), indicating that participants in both the stress and no stress groups successfully learned CS-US contingencies. To examine the role of cortisol response in learning, a 2 (CS valence) × 2 (CS magnitude) × 3 (cortisol response: responders, non-responders, no stress) mixed ANOVA was performed. Cortisol response did not interact with valence or magnitude (all *p*'s > 0.05) with respect to behavioral ratings.

### fMRI results

**Figure 4:**
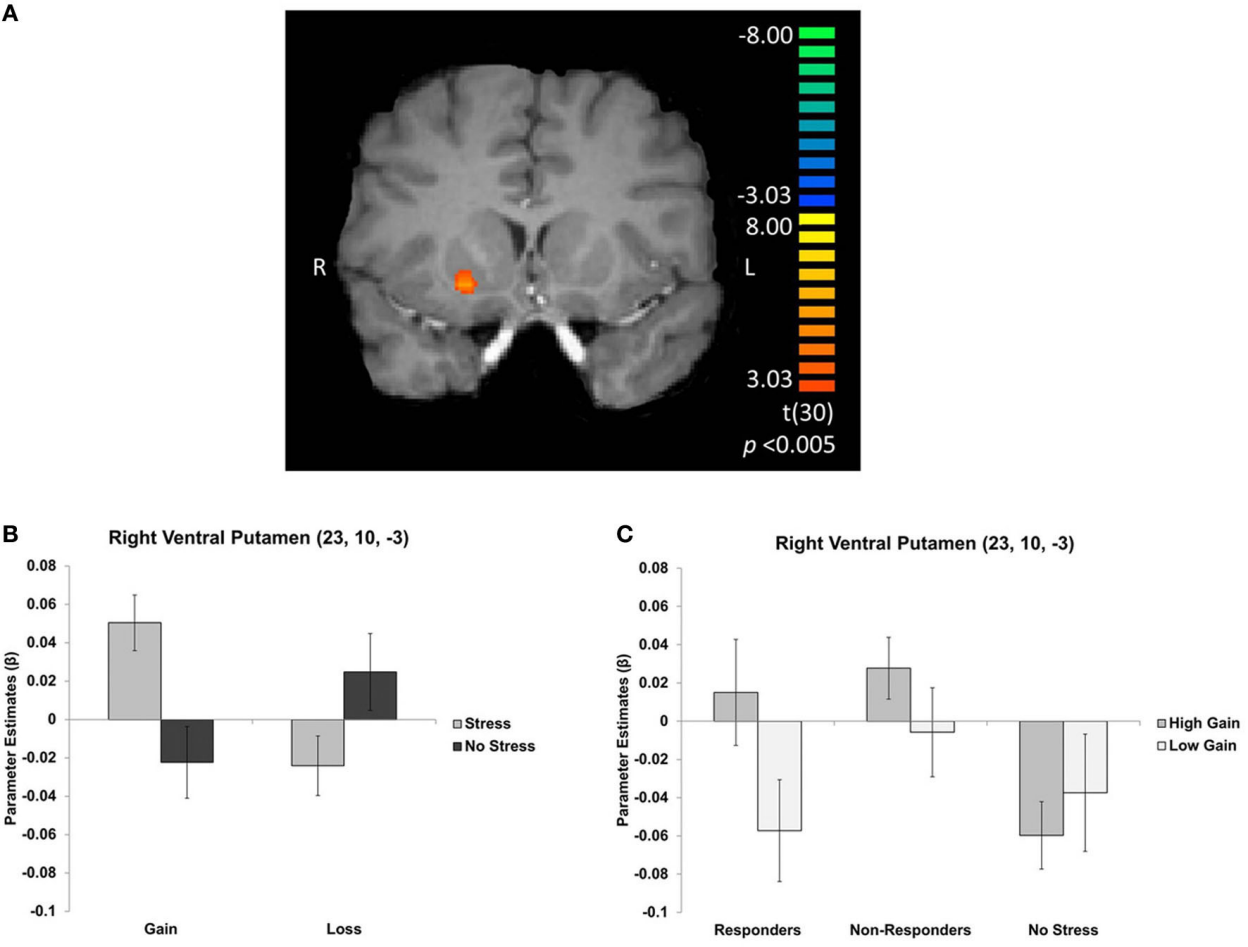
**(A)** Right ventral putamen cluster resulting from an interaction contrast of [(high gain + low loss) − (low gain + high loss)], with the difference in BOLD resulting from this contrast computed along the between-subjects factor of stress group. Graphs depict mean parameter estimates (β) for ROIs in right ventral putamen **(B)** for gains and losses, collapsed across magnitude, and **(C)** for gains only, displayed separately for cortisol responders, non-responders, and no stress participants. Error bars represent s.e.m.

To examine the effects of valence, magnitude, and stress group on neural activation during CS presentation, we performed an interaction contrast of [(low gain + high loss) − (high gain + low loss)] and the difference in BOLD associated with this contrast was computed along the between-subjects factor of stress group (Figure 4A). This contrast yielded significant activity in the ventral striatum, in particular the right ventral putamen (*x*, *y*, *z* = 23, 10, −3), as well as in right medial prefrontal cortex (*x*, *y*, *z* = 14, 40, −6), left parahippocampus (*x*, *y*, *z* = −19, −17, −18), and paracentral lobule (*x*, *y*, *z* = −1, −44, 63). Parameter estimates were then extracted from these regions and *post-hoc t*-tests were performed. All significant clusters are reported in Table 2. Simple contrasts of valence and magnitude within each group are presented in Table 3. Regions of activation resulting from additional contrasts involving valence, magnitude and group differences are reported in Table 4.

**Table 2:** **Regions of activation in an interaction contrast of valence, magnitude, and stress group; *p* < 0.005, corrected**.

**Activated region**	**Laterality**	**Talairach coordinates**			**Voxel count (mm^3^)**	***T*-value**
		***x***	***y***	***z***		
Putamen	R	23	10	−3	177	5.543
Medial prefrontal cortex (BA 32)	R	14	40	−6	152	4.269
Paracentral lobule (BA 5)	L	−1	−44	63	240	4.222
Parahippocampal gyrus (BA 28)	L	−19	−17	−18	136	4.512

BA, Brodmann's area; L, left; R, right.

**Table 3:** **Regions of activation for the contrast between gains and losses and the contrast between high magnitude and low magnitude, in both stress and no stress groups; *p* < 0.005, corrected**.

**Activated region**	**Laterality**	**Talairach coordinates**			**Voxel count (mm^3^)**	***T*-value**
		***x***	***y***	***z***		
**LOSS > GAIN (STRESS GROUP)**						
Parahippocampal gyrus	R	29	−11	−18	259	5.652
**HIGH > LOW (STRESS GROUP)**						
Superior frontal gyrus (BA 10)	L	−40	52	15	406	4.958
**GAIN > LOSS (NO STRESS GROUP)**						
Middle frontal gyrus (BA 8)	R	23	31	39	327	5.162
Cingulate gyrus (BA 31)	R	2	−44	33	325	5.909

Contrasts not listed did not yield any activation that survived cluster correction. BA, Brodmann's area; L, left; R, right.

**Table 4:** **Regions of activation for simple other interaction contrasts; *p* < 0.005, corrected**.

**Activated region**	**Laterality**	**Talairach coordinates**			**Voxel count (mm^3^)**	***T*-value**
		***x***	***y***	***z***		
**STRESS > NO STRESS**						
Lentiform nucleus	L	−22	−8	−6	409	4.410
Parahippocampal gyrus (BA 28)	L	−22	4	−30	204	4.781
Precentral gyrus (BA 6)	L	−46	−8	9	539	4.282
**HIGH > LOW MAGNITUDE (BY STRESS GROUP)**						
Middle frontal gyrus (BA 9)	R	38	28	36	223	4.438
Middle frontal gyrus (BA 9)	L	−49	22	33	268	5.185
**(HIGH GAIN + LOW LOSS) > (LOW GAIN + HIGH LOSS)**						
Superior temporal gyrus (BA 42)	R	59	−29	12	364	3.925
Inferior frontal gyrus (BA 47)	R	53	19	0	298	5.416
Superior temporal gyrus (BA 22)	R	44	−26	3	1106	4.906
Insula (BA 13)	R	41	−5	3	358	5.005
Lingual gyrus (BA 17)	R	8	−89	0	1465	5.214
Lingual gyrus (BA 18)	L	−13	−83	−6	924	5.051
Putamen	L	−31	−14	3	563	5.036

Contrasts not listed did not yield any activation that survived cluster correction. BA, Brodmann's area; L, left; R, right.

In the right ventral putamen, a differential response between the stress and no stress groups was observed, but primarily for the CS predicting high magnitude monetary gains [*t*_(30)_ = 3.574; *p* < 0.005]. Within the stress group this region exhibited an effect of magnitude, with greater BOLD responses for CS predicting high magnitude compared to low magnitude gains [*t*_(15)_ = 3.171; *p* < 0.01]. The no stress group did not exhibit such a difference (Figure 4B). This effect was driven by individual differences in cortisol response in the stress group. Specifically, cortisol responders exhibited sensitivity to gain magnitude [*t*_(6)_ = 3.791; *p* < 0.01], while non-responders did not [*t*_(8)_ = 1.428; *p* > 0.05] (Figure 4C). Thus, the sensitivity to gain magnitude observed in the right ventral putamen of stress group participants was driven by individual differences in circulating cortisol levels (i.e., cortisol responders).

In the medial prefrontal cortex, increased BOLD signal was observed in the stress group as compared to the no stress group in response to the low magnitude gain CS [*t*_(30)_ = 2.940; *p* < 0.01]. Additionally, activity was enhanced in the no stress group for high magnitude loss CS as compared to low magnitude loss CS [*t*_(15)_ = 3.016; *p* < 0.01]; this pattern was not present in the stress group. No differences based on cortisol response were observed.

In the left parahippocampus, *post-hoc t*-tests revealed greater activation in the no stress group as compared to the stress group for high magnitude monetary loss CS *t*_(30)_ = 3.139; (*p* < 0.005). With respect to loss magnitude, opposite patterns manifested in this region between the stress and no stress groups. BOLD signal was greater for low magnitude loss CS as compared to high magnitude loss CS in the stress group, [*t*_(15)_ = 2.750; *p* < 0.05], while this pattern was reversed for the no stress group [*t*_(15)_ = 3.233; *p* < 0.01]. No differences based on cortisol response were observed.

In the paracentral lobule, the stress group showed enhanced sensitivity to loss magnitude, with increased activity to low as compared to high magnitude loss CS [*t*_(15)_ = 3.713; *p* < 0.005]. This pattern was not present in the no stress group. No differences based on cortisol response were observed.

Given the well-documented role of sex in stress research, we conducted an exploratory analysis to examine potential sex differences in the effects of stress on conditioning. Within the stress group, *post-hoc t*-tests were run on all regions that yielded significant effects in the interaction contrast using sex as a factor. No significant sex effects were observed (all *p*'s > 0.05).

## Discussion

In the current study, we sought to understand the effects of stress on the neural correlates underlying Pavlovian conditioning with monetary gains and losses. Specifically, participants learned associations between neutral shapes and monetary outcomes (gain, loss) of varying magnitude (low, high) after exposure to acute stress (or a control condition). Both groups learned the contingencies equally well. Subjective ratings of stress confirmed the efficacy of the stress manipulation. We observed that acute stress rendered the human ventral striatum more sensitive to the magnitude of positively conditioned stimuli, and this may be related to stress-related increases in cortisol. Specifically, as compared to the no stress group, participants in the stress group exhibited differential activity in the ventral striatum, particularly in the right ventral putamen, to cues predicting high as compared to low magnitude gains. Interestingly, this sensitivity to gain magnitude with the stress group was driven by cortisol responders, as non-responders and no stress participants did not show this effect. Taken together, these results provide a preliminary investigation into understanding how individual differences in responses to stress influence neural mechanisms involved in associative learning in humans.

The human striatum is known to be involved in associative learning with both primary (e.g., O'Doherty et al., 2004; see Phelps and LeDoux, 2005, for review) and secondary (Kirsch et al., 2003; Valentin and O'Doherty, 2009; Delgado et al., 2011) reinforcers. Exposure to acute stress has been shown to adversely affect processing in the human striatum during aversive conditioning (Merz et al., 2013b) and outcome processing (Porcelli et al., 2012). We extend these findings by highlighting modulation of ventral striatum activity by acute stress during a Pavlovian conditioning task. In the current study, participants exposed to stress showed greater responses in the ventral striatum to cues associated with monetary gains, and showed enhanced sensitivity to the magnitude of the monetary gain. Interestingly, the locus of this activity was in the ventral putamen, which has been suggested to morphologically change with chronic stress (Dias-Ferreira et al., 2009). Stress is known to shift goal-directed behavior toward habitual responses during instrumental tasks (Schwabe and Wolf, 2009; see Schwabe and Wolf, 2011, for review), and the putamen may play a role in this process (Dias-Ferreira et al., 2009). One potential interpretation of the observed striatum increase in the stress group, therefore, is that it could reflect the initial formation of a habitual representation, which has previously been characterized in the putamen in humans, although at a more dorsal locus (Tricomi et al., 2009).

Indeed, the acquisition of contingencies in the current study occurred quickly, as suggested by subjective ratings. Although no behavioral differences in learning were observed between groups, it is possible that potential differences would only be expressed in the form of instrumental behaviors. In rodents, stress has been found not to behaviorally impair Pavlovian conditioning itself, but rather to lead to deficiencies in Pavlovian-to-instrumental transfer (Morgado et al., 2012)—a measure of the influence of Pavlovian cues on instrumental behavior (for review, see Dickinson and Balleine, 1994). Pavlovian-to instrumental transfer has been associated with increased activity in the striatum, particularly the putamen in humans (Bray et al., 2008; Prevost et al., 2012), and the effect of acute stress on this phenomenon might further elucidate the manner in which stress impacts striatal activity during Pavlovian conditioning.

An alternative interpretation for the increased striatal responses under stress in our conditioning task involves altered processing of learning or value-related signals. Increased responses to prediction errors have been observed in the ventral striatum under stress (Robinson et al., 2013) and it is plausible that increases in responses to a CS of high magnitude involves greater prediction error given that it is the best potential cue possible (similarly, greater decreases observed with a CS of low magnitude, as it does not represent a high magnitude opportunity). However, Robinson et al. (2013) observed increased aversive, rather than appetitive prediction errors under stress, whereas striatal responses in the current study primarily favor increased responses during conditioning with gains, rather than losses. It is possible that, as previously mentioned, examination of acute stress on more instrumental behaviors might allow for better characterization of prediction error signals and shifts in behavioral tendencies.

In addition to acute stress exposure, levels of circulating cortisol have also been shown to affect Pavlovian conditioning (Stark et al., 2006; Merz et al., 2013a). Given that not all participants in our stress group showed increases in cortisol following stress exposure, we separately examined brain activity during conditioning in cortisol responders and non-responders. In the right ventral putamen, cortisol responders exhibited sensitivity to gain magnitude whereas non-responders and no stress participants did not. This suggests that it was cortisol responders who drove the difference between the stress and no stress groups observed in this region. Enhanced acquisition of conditioned responses after acute stress exposure (i.e., classical eyeblink conditioning in rats after restraint and shock stress) is thought to require stress-related release of endogenous glucocorticoids (Beylin and Shors, 2003). Although we did not see differential responses in our behavioral ratings of conditioning between cortisol responders and non-responders, it is possible that modulation of ventral striatum signal during learning by increased cortisol responses manifests during instrumental responses (Morgado et al., 2012), an important question for future research. Converging with the animal literature (e.g., Beylin and Shors, 2003), the current study emphasizes the importance of measuring individual differences in cortisol responses, as levels of this hormone (rather than exposure to stress *per se*) may be responsible for some of the changes observed in the human brain during learning.

Of particular interest is the dissociation between subjective ratings of stress and cortisol responses. While stress group participants reported significantly higher levels of subjective stress than did the no stress group, several participants in the stress group did not show elevated cortisol levels. In fact, subjective ratings of stress were not significantly correlated with cortisol responses in the current study (all *p*'s > 0.05), as has been the case in several previous studies (see Campbell and Ehlert, 2012, for review). In research as well as in clinical settings, therefore, it may be valuable to examine both physiological and subjective measures of stress in order to fully characterize the stress experience.

It is crucial to note that acute stress affects not only the hypothalamic-pituitary-adrenal (HPA) axis, but also the sympathetic branch of the autonomic nervous system (ANS; for review see Ulrich-Lai and Herman, 2009). Recent research suggests that concurrent HPA and sympathetic ANS activation may be required for stress-induced shifts toward habitual responses in instrumental conditioning paradigms (e.g., Schwabe et al., 2010, 2012). While the HPA response and associated glucocorticoid release was measured in the current study through collection of salivary cortisol, we did not measure sympathetic ANS activation (e.g., via collection of salivary alpha amylase; Rohleder et al., 2004), a limitation of our study. Thus, it is plausible that the results presented here are not purely cortisol driven and rather reflect an interaction between both the HPA and sympathetic ANS associated adrenergic activity. Future studies of acute stress effects on learning processes should incorporate measures that allow dissociation between the roles of cortisol and adrenergic systems.

In order to further explore this hypothesis, we turned to participants from the no stress group that showed at least a 15.5% increase in cortisol above baseline. In Miller et al. (2013), a no stress (i.e., placebo) group “responder” rate of 28.7% was observed. Consistent with these results, four participants in the no stress group in the current study met this criterion (30.8%; mean increase = 0.056 μg/dL). To examine whether or not observed differences in striatal sensitivity to gain magnitude (as in stress group responders but not stress group non-responders) was associated with increased cortisol alone vs. a combination of the stress manipulation *and* increased cortisol, we performed an analogous analysis on the no stress group “responders” and “non-responders” only. Within the no stress group, the striatum was *not* sensitive to magnitude in the gain and loss domains regardless of “responder” or “non-responder” status (all *p*'s > 0.05). Although it is uncommon to break up a no stress group into “responders” and “non-responders,” this analysis does suggest that our cortisol responder-based striatum finding is unique to participants who underwent the stress manipulation. Assuming that participants in the no stress group experienced no significant stress-associated sympathetic ANS activation in this study, it is plausible that concurrent HPA and sympathetic ANS activity is responsible for stress group differences observed in response to gain magnitude in the striatum.

The type of stimuli used in our task differed significantly from stimuli used in previous research on stress and conditioning. The current study utilized both gain and loss stimuli, whereas past fMRI studies of stress and conditioning have relied on fearful aversive stimuli. We also used monetary reinforcers, which are not inherently appetitive or aversive (unlike primary reinforcers, such as food and shock). Therefore, we did not expect to see activity in the amygdala as had been observed in previous studies (Merz et al., 2013a,b) given that this region is not necessarily crucial for aversive conditioning with monetary reinforcers in certain contexts (Delgado et al., 2011).

In addition to ventral putamen, a key area of interest in the investigation, other regions that showed an interaction of stress, valence, and magnitude during Pavlovian conditioning are worthy of discussion. We observed enhanced activation of the medial prefrontal cortex, with peak activation in the anterior cingulate cortex (BA 32), in the presence of low magnitude monetary gain CS in the stress group as compared to the no stress group. Given its involvement in the representation of CS during aversive conditioning (Büchel et al., 1998), it was unsurprising to find that under stress the cingulate cortex exhibited dampened sensitivity to loss magnitude. However, the region of anterior cingulate cortex observed in previous conditioning research (e.g., Büchel et al., 1998) is more dorsal than that observed in the current study. Therefore, the observed changes in medial prefrontal cortex activation under stress may actually reflect changes in perceived value of the presented stimuli (see Rushworth and Behrens, 2008, for review). Additionally, we found that acute stress affected the processing of CS in the parahippocampus, wherein activity was diminished in response to high magnitude losses. Past research suggests that parahippocampal activity correlates with awareness of aversive CS-US contingencies during conditioning (Carter et al., 2006); therefore, the observed differences in the parahippocampus may reflect an effect of stress on contingency awareness. While we had no *a priori* hypotheses regarding the paracentral lobule, it has been found that stress enhances activity in resting state brain networks, including the sensorimotor network which contains the paracentral lobule (Soares et al., 2013).

Sex has played a role in previous studies of stress and conditioning. In particular, research indicates that stress differentially affects conditioning in males and females in both rodents (Wood and Shors, 1998; Wood et al., 2001) and humans (Duncko et al., 2007). Recent neuroimaging work suggests that certain brain regions may be differentially modulated by stress in males and females during fear conditioning (Merz et al., 2013b). These results have been largely variable and seem to depend on a number of factors such as the type of stressor used. In the current study, we did not observe any effects of sex on brain activity in the stress group. Potential sex effects may have been hard to discern, however, given that our study was not aimed at examining sex differences where a significantly larger sample size may be necessary. Nonetheless, it is important to acknowledge that sex is an important factor that may influence the manner in which stress affects learning processes.

The current study has several additional limitations. First, the cortisol responder analysis is based on a small sample of seven responders and nine non-responders; therefore, our conclusions will benefit from replication and extension. Additionally, our cortisol manipulation was not completely successful, as the AUC_I_ measurement did not reach significance. While the current study examined the manner in which acute stress influences conditioning, it is important to note that chronic stress may also play a role. We did not examine the effects of preexisting chronic stress on Pavlovian conditioning; however, this is an important direction for future studies. It will also be useful to more carefully consider the participant pool in future work on this topic. For instance, the current study did not exclude chronic smokers, yet recurrent nicotine exposure can potentially result in elevated cortisol levels as well as diminished sensitivity of the cortisol response (see Kirschbaum and Hellhammer, 1994 for review). We also did not exclude females taking oral contraceptives, yet research suggests that oral contraceptives may lead to a diminished cortisol response following stress exposure (Kirschbaum et al., 1995). Given these limitations, our study can be considered a preliminary investigation into a very interesting topic and a good initial step that informs future studies.

In sum, the current study found that stress affects the neural correlates of Pavlovian conditioning in regions such as the ventral striatum, particularly the ventral putamen, and that the observed differences are in part related to changes in circulating cortisol. This research adds to a body of existing literature that is working toward comprehension of how stress affects learning more generally. Given that stress is known to increase drug craving (e.g., Sinha et al., 2005) and can potentiate habitual behaviors (Dias-Ferreira et al., 2009; Schwabe and Wolf, 2009), the current study has implications for better understanding how acute stress affects the processing of—and subsequent behavior resulting from—drug-relevant CS in the environment. In particular, this study aids in understanding of how stress may lead to drug abuse via stress-related modulation of Pavlovian conditioning. Our data suggest a preliminary link between individual responses to stress and enhanced sensitivity in the human striatum in response to rewarding conditioned stimuli.

### Conflict of interest statement

The authors declare that the research was conducted in the absence of any commercial or financial relationships that could be construed as a potential conflict of interest.
